# Preparation of ε-Fe(Si)_3_N Powder Using a Salt Bath Nitriding Reaction and a Study on the Soft Magnetic Properties of the Powder

**DOI:** 10.3390/ma12020228

**Published:** 2019-01-11

**Authors:** Yuhua Xu, Zhenghou Zhu, Hui Zhao, Jia Zhou

**Affiliations:** 1 School of Materials Science & Engineering, Nanchang University, Nanchang 330031, China; xuyuhua@ncu.edu.cn; 2 Institute of Space Science and Technology, Nanchang University, Nanchang 330031, China; candyzhaohui@126.com (H.Z.); zhoujia@ncu.edu.cn (J.Z.)

**Keywords:** FeSi alloy powder, salt bath nitriding reaction, soft magnetic properties, magnetization temperature dependence

## Abstract

In this paper, a single phase ε-Fe(Si)_3_N powder was successfully synthesized through the salt bath nitriding reaction method. The flaky FeSi alloy powder was used as the iron source, and non-toxic CO(NH_2_)_2_ was used as the nitrogen source. The nitridation mechanism, the preparation technology, the soft magnetic properties, and the magnetization temperature dependence of the powder were studied. The research result showed that ε-Fe(Si)_3_N alloy powders were synthesized in a high temperature nitrification system after the surface of flaky FeSi alloy powders were activated by a high-energy ball mill. The optimum nitriding process was nitridation for 1 h at 550 °C. The ε-Fe(Si)_3_N powder had good thermal stability at less than 478.8 °C. It was shown that ε-Fe(Si)_3_N powder has good soft magnetic properties, and the saturation magnetization of the powder was up to 139 emu/g. The saturation magnetization of ε-Fe(Si)_3_N powder remains basically constant in the temperature range of 300–400 K. In the temperature range of 400–600 K, the saturation magnetization decreases slightly with the increase of temperature, indicating that the magnetic ε-Fe(Si)_3_N powder has good magnetization temperature dependence.

## 1. Introduction

Early on, nitrogen was introduced into iron-based materials. Its main purpose was to improve corrosion resistance and the mechanical properties of materials, such as surface hardness, wear resistance, fatigue strength, etc. [1,2,3]. In 1972, Kim and Takahashi discovered for the first time that α″-Fe_16_N_2_ has a “giant magnetization” phenomenon [4]. Since then, many researchers have moved from studying its mechanical properties to studying its magnetic properties. According to the ratio of nitrogen, the Fe–N system includes iron saturated with nitrogen α-Fe(N), α′-Fe-N martensite, and several Fe–N interstitial compounds with nitrogen ordering (ξ-Fe_2_N, ε-Fe_3_N, γ′-Fe_4_N, and α″-Fe_16_N_2_) [5]. FeN and Fe_2_N are weakly magnetic, and α″-Fe_16_N_2_ is more magnetic. However, because α″-Fe_16_N_2_ is metastable, it is difficult to synthesize and has poor thermodynamic stability. Above 200 °C, it decomposes into α-Fe and γ′-Fe_4_N, which greatly limits its application. Although the magnetism of Fe_3_N is not as good as that of α″-Fe_16_N_2_ and α-Fe (the magnetization is 220 emu/g, and the magnetic polarization is about 2.2 T), it has better thermodynamic stability than that of α″-Fe_16_N_2_ and can be preserved at room temperature. Compared with pure iron, it has the advantage of good oxidation resistance. It also has good corrosion resistance and mechanical properties, high saturation magnetization, high magnetic moment, magnetic permeability, and low coercivity. Hence, it has become a hot topic for research [6,7,8].

At present, the preparation methods of preparing ε-Fe_3_N generally include magnetron sputtering [9,10], gas reduction-nitriding [11,12], the solid-gas reaction method [3,13,14,15], the sol-gel method [16,17,18], etc. Solid-gas nitriding is the main method, where pure iron powders are reacted with ammonia or a mixed atmosphere of ammonia and hydrogen under a certain temperature to synthesize iron nitride. As an example, Lian et al. and Ren et al. used pure Fe powder as an iron source and got ε-Fe_3_N [13,14] using the solid-gas nitriding method. They held it for 3–7 h under certain temperature conditions and then cooled it with liquid nitrogen or natural cooling. ε-Fe_3_N was first synthesized using the solid-gas reaction method by Zhenghe Hua et al. and Wenxu Yin et al. after preparing the precursor with the sol-gel method [15,16]. Wu et al. used a chemical co-precipitation method to prepare the Fe_3_O_4_ nanoparticles, then the dried Fe_3_O_4_ nanoparticles were treated in an H_2_ + NH_3_ mixed gas flow (with a volume ratio of H_2_/NH_3_ of 1:2) at 400–900 °C for 6 h to get ε-Fe_3_N [5]. Kurian and Gajbhiye firstly decomposed the ferric citrate (Aldrich, 99.9%) to fine particles of α-Fe_2_O_3_, then the nitridation of these particles was carried out in a quartz tubular furnace under flowing NH_3_ gas at 873 K for 5.5 h to get ε-Fe_3_N [3]. It is evident that the existing Fe_3_N powder preparation technology has problems, such as a long nitriding time, a complicated process, high equipment requirements, and low efficiency. These seriously restrict the development of ε-Fe_3_N powder preparation technology.

In view of this, we reported a new preparation technology for powder nitriding—the salt bath nitriding reaction method. This preparation technology has characteristics of simplicity, high effectiveness, operability, and a low demand for experimental equipment. In this paper, the ε-Fe(Si)_3_N powder, with excellent magneto-caloric effects and soft magnetic properties, was successfully synthesized using a rapid and efficient nitriding method with a controlled treatment process. The flaky FeSi alloy powder was used to replace pure iron powder as the iron source, and it was immersed in the molten atomic liquid system containing N for the reaction. Fe(Si)_3_N powders with excellent soft magnetic properties and temperature dependence were successfully prepared by controlling the nitriding process to achieve a fast and efficient nitriding method. The ε-Fe(Si)_3_N powder can be applied in a magnetic powder core inductor with a high-frequency circuit because its excellent soft magnetic properties and excellent temperature dependency enable this material to be applied to electronic components under special conditions.

In this paper, two issues of the nitriding of powder were successfully solved. The first is that the alloy powders were difficult to fully nitrify, and the nitriding efficiency was low. Flaky FeSi alloy powders after high-energy ball milling not only possess a large surface area, but also high surface activity. This greatly enlarges the contact area and produces the effect of “double-sided simultaneous nitriding” during nitriding. Thus, the full nitriding time of the powders is greatly shortened, and the efficiency of the nitriding process is greatly improved. Secondly, the Si element increases the entropy values of the powder and reduces the difficulty of the nitriding reaction.

## 2. Materials and Methods

### 2.1. Preparation Technology of FeSiN Powder

**Figure 1:**
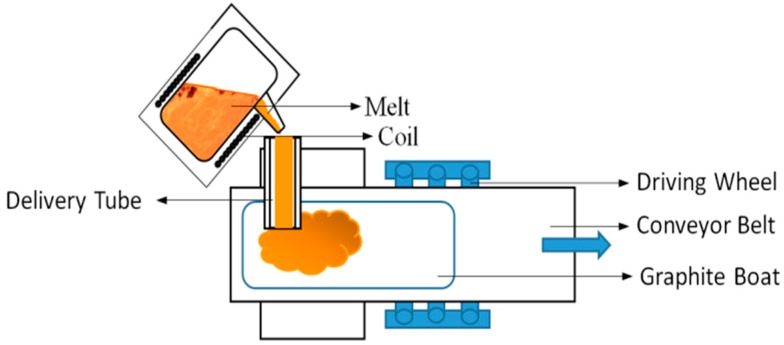
Schematic diagram of FeSi master alloy casting.

FeSi master alloy was prepared using medium-frequency induction melting. The thin sheet mother alloy was prepared with a moving graphite boat in a “3D printing” manner. The technological process of preparation is shown in Figure 1. The master alloy was milled by high-energy vibration ball milling and then the flaky FeSi alloy powder, with a sheet width of 75–150 μm and 45–75 μm, was obtained by sieving.

The nitrogen salt reaction system was composed of nontoxic CO(NH_2_)_2_, NaCl, Na_2_CO_3_, and KOH as raw materials, according to a certain weight ratio. The system was heated to 570 °C and melted into liquid. Then, the temperature of the system was adjusted to the nitriding reaction temperature.

**Figure 2:**
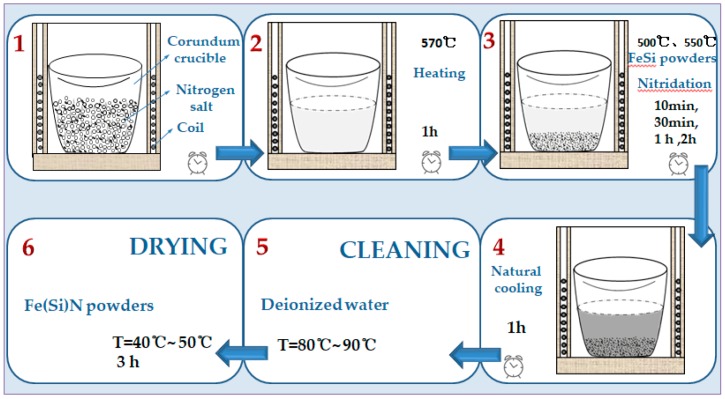
Scheme of the FeSiN powder preparation process.

The flaky FeSi alloy powder was immersed into the liquid reaction system, and the reaction times used were 10 min, 30 min, l h, and 2 h. After the natural cooling of the product, it was washed several times with deionized water at 80–90 °C to remove impurities on the surface of the powder. Then, it was dried at 40–50 °C to obtain a final product. The preparation process is shown in Figure 2.

### 2.2. Characterization

The crystalline structure of the alloy powders was analyzed using X-ray diffraction (XRD, PANalytical EMPYREAN, PANalytical B.V., Almero, Netherlands) with Cu-Kα radiation at 40 kV and 40 mA. The morphology of the powders was observed using a cold field emission scanning electron microscope (FESEM, JSM-6701F, JEOL Ltd. Tokyo, Japan). Magnetic properties were tested using a Physical Property Measurement System (PPMS-9, Quantum Design, San Diego, CA, USA) equipped with a 9 T vibrating sample magnetometer. Thermal stability analysis was recorded on a simultaneous thermal analyzer (TGA/DSC STA449F5, Netzsch, Selb, Germany) from room temperature to 800 °C at a 10 °C/min heating ramp with continuous nitrogen flow. Micro-area element distribution analysis was analyzed by using an electron probe micro-analyzer (EMPA, JXA-8230, JEOL Ltd. Tokyo, Japan). The test conditions were a voltage of 15.0 kV, test current of 5.078 × 10^−7^ A, and micro-area magnification of 50,000×.

## 3. Study of the Nitridation Mechanism

**Figure 3:**
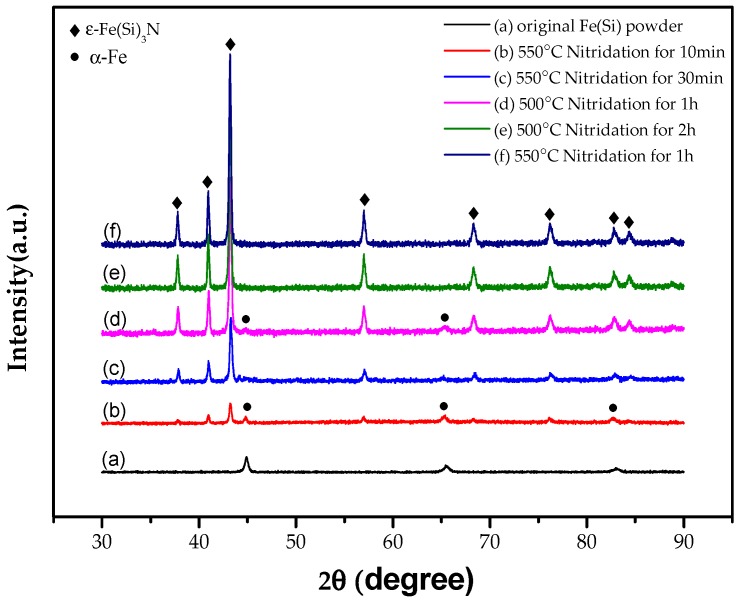
XRD spectra of different powder samples. (**a**) Flaky FeSi alloy powder. (**b**–**f**) Nitriding samples at different temperatures and times.

In order to investigate the effect of the salt bath nitriding reaction preparation process on the nitriding of powder, this paper studied the influences of nitriding temperature (i.e., 500 or 550 °C) and nitriding time (i.e., 10 min, 30 min, 1 h, or 2 h) on powder nitride. The phase structures of the flaky FeSi powder before and after nitridation are shown in Figure 3.

Figure 3 shows the original flaky FeSi powder which was successfully prepared by medium frequency melting and high-energy vibration ball milling and the nitriding of samples at different temperatures and times. It can be seen in Figure 3b–d that the peak of the α-Fe phase still exists in the XRD spectra, indicating that the flake alloy powder nitride prepared at 550 °C for 10 min and 30 min and prepared at 500 °C for 1 h is not enough to make the powder fully nitrided. The XRD spectra shown in Figure 3e show that when the nitriding temperature of the alloy powder remains unchanged at 500 °C and the nitriding time is prolonged to 2 h, there is no α-Fe phase, and the alloy powder was fully nitrided. When keeping the nitriding time at 1 h and increasing the nitriding temperature to 550 °C, the α-Fe phase was not found, as shown in Figure 3f. It was evident that prolonging the nitriding time or increasing nitriding temperature was beneficial to the full nitridation of the flaky FeSi alloy powder.

**Figure 4:**
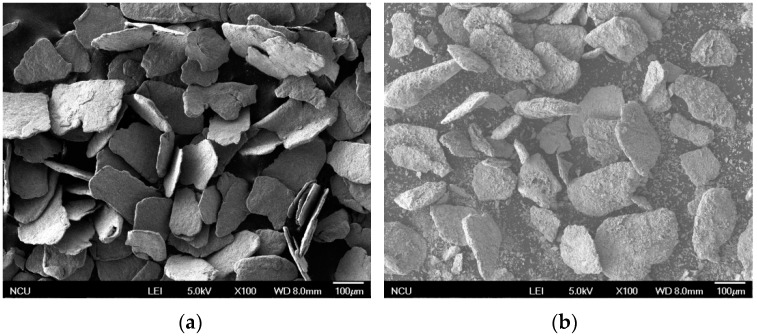
SEM images of FeSi alloy powders before and after nitriding. (**a**) SEM image of flaky FeSi alloy powder. (**b**) SEM image of flaky FeSi alloy powder after nitriding.

Figure 4 shows the contrast of FeSi alloy powders before and after nitriding. It can be seen from Figure 4a that the FeSi alloy powder after high-energy vibration ball milling has a sheet-like irregular morphology with a thickness of about 10 μm. This is because the FeSi alloy particles are repeatedly and violently collided, extruded, and welded in high-energy vibration ball milling, which leads to small flat sheet powder forming. As the ball mill continues, the particles undergo plastic deformation, and internal defects (dislocations, vacancies, etc.) increase under the severe impact, which leads to the continuous refinement of the particles into powder of different particle sizes in thin sheets.

The microstructure of the powder at 550 °C nitridation for 1 h was analyzed. The morphology of the powder after nitriding is shown in Figure 4b. It can be seen from the figure that the shape of the powder particles after nitriding is basically unchanged, but the surface of the powder becomes rough and uneven. This is caused by the reaction and diffusion of nitrogen atoms with the surface of the powder during the nitriding process. When an element penetrates from the metal surface to the inside through diffusion, an intermediate phase (which could be another solid solution) may be formed in the metal surface layer as the diffusion proceeds if the content of the diffusion element exceeds the solubility of the base metal. This phenomenon in which a new phase is formed by diffusion is called reaction-diffusion or phase change diffusion [19].

The nitriding process of flaky FeSi powders is a reaction-diffusion process where flaky FeSi powders are immersed into a solution containing the N atom system. Firstly, the active N atoms in the system react with the surface of the alloy powder at a high temperature. The nitriding reaction process is as follows:

CO(NH_2_)_2_ + Na_2_CO_3_ → CNO^−^ + H_2_O + NH_3_↑ + CO_2_↑

CNO^−^ → CO_3_^2−^ + CN^−^ + CO + [N]

Fe(Si) + [N] → N dissolves in α-Fe(Si)

3Fe(Si) + [N] → Fe(Si)_3_N

CN^−^ + [O] → CNO

At the same time as the reaction, the N atoms diffuse through the defects that were generated during the high-energy ball milling of the powder. Secondly, the N atoms in the system are excited by the temperature and the system compound. If the chemical potential of N in the solution of the system is high enough, then the nitrogen atoms gradually diffuse from the surface of the powder into the interior of the powder.

According to the Arrhenius-type equation [19], the diffusion coefficient (D) is related to the activation energy.

(1) $$D=D_{0}\exp\;(-Q/\mathrm{RT})$$

where D_0_ is the pre-exponential term, Q is the activation energy (in units of cal/mol) for the species under consideration, R is the gas constant, and T is the absolute temperature (K).

During the nitridation reaction, the diffusion of nitrogen atoms is mainly affected by temperature, defects, and N atomic chemical potential or $\mu_{i}$ [19]. The kinetics of the process of diffusion is strongly dependent on temperature. When the temperature of the nitrification system increases, the thermal energy that was supplied to the diffusing atoms permits the atoms to overcome the activation energy barrier, and they more easily to move to new sites. Therefore, the flux of atoms increases, as well. The XRD spectra also showed that the nitriding effect was better when the nitriding reaction time was shortened from 2 to 1 h and the nitriding temperature was raised from 500 to 550 °C. The saturation magnetization of the product was slightly higher than that of the product nitrided at 500 °C for 2 h, which was 139 emu/g. The detailed test results of the saturation magnetization of powders under different nitridation conditions are shown in Table 1. 

The chemical potential $\mu_{i}$ of the N atom in the nitrogen salt system indicates the driving force for diffusion. Under the action of the thermal activation energy, the N atoms in the system maintain a high enough chemical potential and diffuse, in depth, continuously and divergently along the defects in the powder. This continues until the chemical potential gradient $\left(\frac{\partial \mu_{i}}{\partial x}\right)$ is 0 in the nitrided layer and there is no driving force for diffusion. The diffusion of nitrogen atoms cannot continue, and the nitriding of powders is complete.

**Figure 5:**
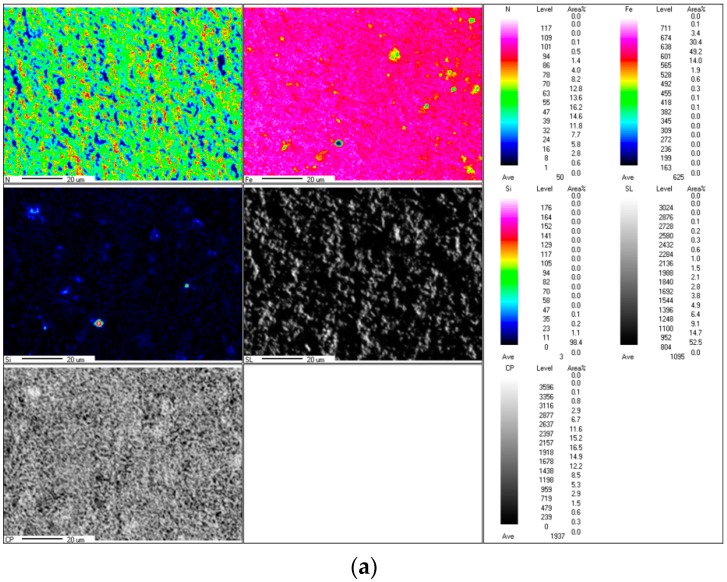

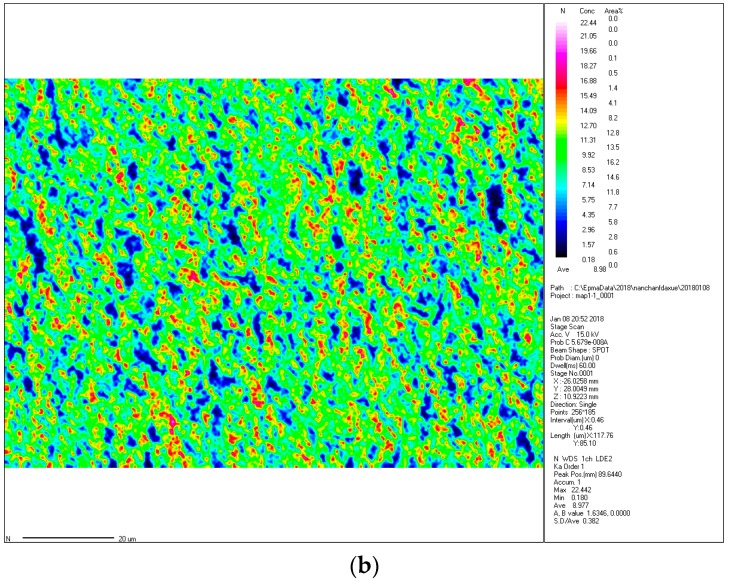
The ε-Fe(Si)_3_N powder element distribution (**a**) Fe, Si and N element distribution. (**b**) N element distribution.

The nitrogen content was analyzed by using an electron probe microanalyzer (EPMA), which used a focused electron beam irradiated onto a small area of the surface of the sample to excite characteristic X-rays of the Fe element and the N element wavelength (energy) in the sample. Figure 5a,b depicts micro-area distribution maps of the Fe and N elements in the ε-Fe(Si)_3_N powder, respectively. In Figure 5, it is evident that the iron content is relatively large and well-distributed, and the Fe element cluster enrichment region is substantially the same as the N element cluster barren region. Combined with the above XRD pattern, it can be concluded that the Fe element takes the N element as the nucleation core and forms the ε-Fe(Si)_3_N phase in the nitriding process.

**Figure 6:**
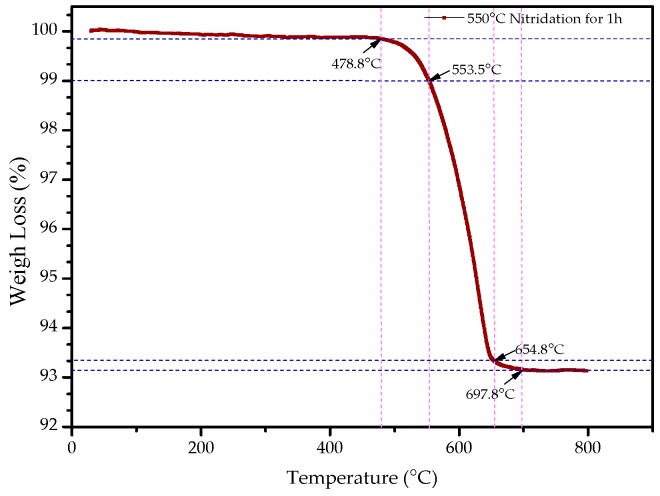
TG curve of ε-Fe(Si)_3_N.

The thermal decomposition of nitrides should be considered as the weakened metal N bond broken by heat motion and N atoms combined into the strong nitrogen molecule. In order to better understand the thermal stability of nitriding powders, the thermal stability test was carried out using a synchronous thermal analyzer. Thermogravimetric analysis (TG) is a thermal analysis technique for measuring the relationship between the mass of the sample and the temperature change. Figure 6 shows the thermal analysis curve of the ε-Fe(Si)_3_N powder.

It can be seen from Figure 6 that the weight of ε-Fe(Si)_3_N powders changes gradually at first, then loses weight sharply. Eventually, it tended to become stable with the increase of temperature. When the ε-Fe(Si)_3_N powders were raised from room temperature to 478.8 °C, their weights did not change. The turning point temperature of weightlessness was 478.8 °C. From the turning point temperature to 553.5 °C, its weightlessness ratio was 1%, then the weight decreased sharply. When the temperature was raised to 654.8 °C, the weightlessness ratio was about 6.5%, and the TG curve began to stabilize. Finally, the weightlessness of the powders was almost complete, and the TG curve showed a steady trend at a temperature of 697.8 °C. This is because the nitrogen in the ε-Fe(Si)_3_N powders gradually formed nitrogen gas and escaped with the increase of temperature, which made the weight of the powders slowly decrease. The results show that the properties of the powders themselves basically did not change under the working environment before the turning point temperature (478.8 °C) of weightlessness. Because the weight was basically undergoing no loss, no nitrogen atom formed nitrogen gas to escape and made chemical structure changes in Fe(Si)_3_N.

## 4. Results and Discussion

### 4.1. Magnetic Properties of the FeSiN Powder

**Figure 7:**
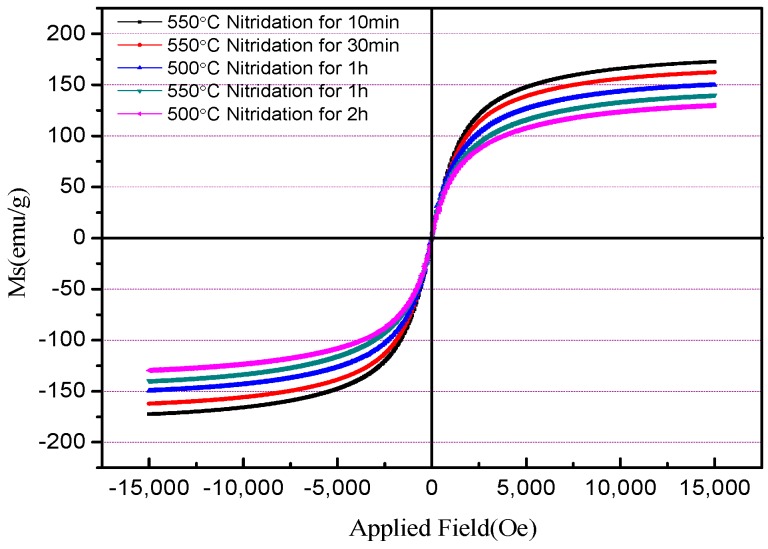
Magnetic hysteresis loop of the Fe(Si) nitride powder at room temperature.

Figure 7 shows the hysteresis loops of FeSiN powders tested by a Physical Property Measurement System (PPMS-9, Quantum Design, USA) equipped with a 9 T vibrating sample magnetometer.

The highest saturation magnetization (M_S_) refers to maximum magnetization of magnetic materials when it is magnetized in an external magnetic field. It can be seen from Figure 7 that the highest saturation magnetization (M_S_) value of the alloy powders is obtained after nitridation for 10 min. The saturation magnetization of the nitride powders decreases with the increase of nitriding temperature and nitriding time. The relationship between nitriding temperature, nitriding time, and saturation magnetization of FeSiN powders is shown in Table 1. For powder obtained after nitridation at 500 °C for 1 h, the saturation magnetization was 149 emu/g, and the saturation magnetization of the powder decreased to 129 emu/g when the nitriding time was increased to 2 h. For powder obtained after nitridation at 550 °C for 10 min, the saturation magnetization was as high as 172 emu/g. When the nitriding time is extended to 30 min and 1 h, the saturation magnetization was 161 and 139 emu/g, respectively. This is because the nitrification reaction of the powder is more complete at increased nitriding temperatures and at prolonged nitriding times. Corroboration of this can also be obtained from the XRD pattern in Figure 2. An α-Fe phase still exists when the powder is nitrided at 550 °C for 10 min and 30 min and at 500 °C for 1 h. However, there is no α-Fe phase at 550 °C nitridation for 1 h and 500 °C nitridation for 2 h. It is indicated that the nitrogen atom leaks into the iron base as a non-magnetic substance. The number of non-magnetic nitrogen atoms in the iron-based powder increases, and the content of ferromagnetic materials decreases relative to the increase of nitriding temperature and the prolongation of nitriding time. The powder is nitrided more completely and fully so that the saturation magnetization of the sample after nitriding becomes increasingly lower. However, the Ms of the powder is still as high as 139 emu/g at 550 °C nitridation for 1 h. It shows that the ε-Fe(Si)_3_N powder synthesized using the salt bath nitriding reaction has good soft magnetic properties, which lays a foundation for the preparation of a magnetic powder core with excellent properties.

### 4.2. Magnetization Temperature Dependence of ε-Fe(Si)_3_N Powder

All ferromagnetic materials show a decrease of magnetism-related properties, such as magnetization, with increasing temperature, due to the destruction of magnetic order. Therefore, it is particularly important to study the temperature dependence of the magnetic ε-Fe(Si)_3_N powder. However, an important reference for magnetization temperature dependence is the curve of saturation magnetization and temperature.

**Figure 8:**
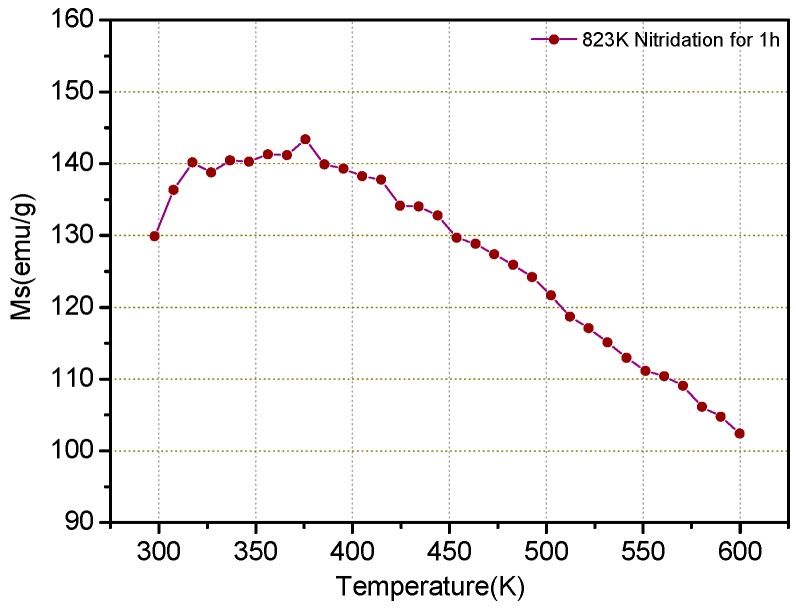
Saturation magnetization and temperature (Ms-T) curve of ε-Fe(Si)_3_N.

Figure 8 shows the relationships between the saturation magnetization (Ms) and the temperature (T) for the ε-Fe(Si)_3_N powders under an applied field up to 10 kOe.

It can be seen from Figure 8 that the saturation magnetization of the powders first increases, then tends to be constant in a certain temperature range. It then gradually decreases with the increase of temperature. The saturation magnetization of ε-Fe(Si)_3_N powder is 129.91 emu/g at 300 K. When the temperature rises to 317 K, the saturation magnetization increases to 140.18 emu/g. Then, in the range of 317–395 K, it basically holds steady. The vibration is a saturation magnetization of about ±1 emu/g, and the maximum amplitude increase of the saturation magnetization is 8.8%. In the range of 400 to 600 K, the saturation magnetization decreases from 141.29 to 102.40 emu/g with increasing temperature, which is obviously superior to that of Fe_3_N powders [20,21].

## 5. Conclusions

The FeSiN powder was prepared using the salt bath nitriding reaction method with flaky FeSi alloy powder as the iron source and non-toxic CO(NH_2_)_2_ as the nitrogen source, and the different nitrogen contents of FeSiN powder can be obtained through changing the nitridation temperature and nitridation time. Under optimal conditions (i.e., a nitriding temperature of 550 °C and nitriding time of 1 h), the single phase ε-Fe(Si)_3_N powder was synthesized with a saturation magnetization as high as 139 emu/g.

The thermal stability and magnetization temperature dependence of the single phase ε-Fe(Si)_3_N powders were investigated. It was found that the ε-Fe(Si)_3_N powder experienced basically no weight loss before 478.8 °C, indicating that Fe(Si)_3_N did not decompose. Therefore, the ε-Fe(Si)_3_N powder has good thermal stability. The saturation magnetization of the ε-Fe(Si)_3_N powder remains basically constant in the temperature range of 300–400 K. In the temperature range of 400–600 K, the saturation magnetization decreases slightly with the increase of temperature, indicating that the magnetic ε-Fe(Si)_3_N powder has good magnetization temperature dependence.

## Figures and Tables

**Table 1:** Relationship between nitriding temperature, nitriding time, and saturation magnetization of FeSiN powders.

Nitriding Temperature (T)	Nitriding Time (t)	Saturation Magnetization (Ms)
550 °C	10 min	172 emu/g
550 °C	30 min	161 emu/g
500 °C	1 h	149 emu/g
550 °C	1 h	139 emu/g
500 °C	2 h	129 emu/g
